# Inhibition Roles of Calcium in Cadmium Uptake and Translocation in Rice: A Review

**DOI:** 10.3390/ijms241411587

**Published:** 2023-07-18

**Authors:** Junli Liu, Xiaoyu Feng, Gaoyang Qiu, Hua Li, Yuan Wang, Xiaodong Chen, Qinglin Fu, Bin Guo

**Affiliations:** 1 State Key Laboratory for Managing Biotic and Chemical Threats to the Quality and Safety of Agro-Products, Institute of Environment, Resource, Soil and Fertilizer, Zhejiang Academy of Agricultural Sciences, Hangzhou 310021, China; liujunli@zaas.ac.cn (J.L.); 12214079@zju.edu.cn (X.F.); qiugy@zaas.ac.cn (G.Q.); lihua@zaas.ac.cn (H.L.); wangyuan@zaas.ac.cn (Y.W.); chenxiaodong@zaas.ac.cn (X.C.); fuql@zaas.ac.cn (Q.F.); 2 Key Laboratory of Environment Remediation and Ecological Health, Ministry of Education, College of Environmental Resource Sciences, Zhejiang University, Hangzhou 310058, China

**Keywords:** antagonism of ions, cadmium, calcium, rice, translocation

## Abstract

Cadmium (Cd) contamination in rice grains is posing a significant threat to global food security. To restrict the transport of Cd in the soil-rice system, an efficient way is to use the ionomics strategy. Since calcium (Ca) and Cd have similar ionic radii, their uptake and translocation may be linked in multiple aspects in rice. However, the underlying antagonistic mechanisms are still not fully understood. Therefore, we first summarized the current knowledge on the physiological and molecular footprints of Cd translocation in plants and then explored the potential antagonistic points between Ca and Cd in rice, including exchange adsorption on roots, plant cell-wall composition, co-transporter gene expression, and transpiration inhibition. This review provides suggestions for Ca/Cd interaction studies on rice and introduces ionomics research as a means of better controlling the accumulation of Cd in plants.

## 1. Introduction

Rice is one of the most critical staple crops in the world, serving as a primary source of sustenance for over half of the world’s population. It provides millions of people in developing countries with basal carbohydrates, vitamins, and minerals. However, the widespread occurrence of cadmium (Cd) pollution in soil–rice ecosystems has had a negative impact on the production of safe rice, and is particularly acute in China [1]. Rapid industrial development and inadequate environmental protection over the past thirty years have caused widespread Cd pollution, particularly in areas surrounding smelting facilities and metal-mining sites. In China, millions of hectares of arable land have been contaminated by Cd, mainly by the irrigation of industrial wastewaters [2]. Meanwhile, rice is an efficient crop for the uptake of Cd from contaminated paddy soils, leading to the accumulation of excessive levels of Cd in the grains. Consumption of the contaminated rice grains has become a major source of Cd exposure for the general population. This raises serious concerns about food safety in China, where the quantity and quality of the food supply is already a pressing issue [3].

The application of lime is an efficient means of controlling the translocation of soil Cd into rice grains. Lime materials, including quicklime and limestone, are low-cost, and are easily accepted by farmers, and have a significant effect on increasing soil pH and reducing available Cd of soil [4]. Meanwhile, the appropriate application of lime can also recover the Ca content in acidified soil. It has long been known that calcium (Ca) is an essential element for plants, and is crucial in maintaining cell-wall structure, membrane stability, and participating in plant signal transduction [5]. Since Ca and Cd have similar ionic radii, these two elements may exhibit antagonistic effects at multiple levels in plants. Maintaining and supplementing a certain amount of Ca nutrition may also improve the Cd resistance of rice.

However, to date, the antagonistic mechanism between Ca and Cd in plants, especially for rice, has not been thoroughly understood. According to the mode of Ca and Cd, their uptake and translocation may be joined in the following aspects, including exchange absorption on root, plant cell-wall composition, co-transporter gene expression, and transpiration inhibition. Thus, this review systematically elaborates on the migration process of Cd in the soil–plant system, and then discusses the possible mechanisms of Ca in inhibiting Cd translocation in rice plants. The aim of the paper was to provide theoretical and practical support for the utilization of Ca materials in preventing Cd accumulation in rice and improving food safety.

## 2. Pathways of Cadmium in Soil–Plant System

### 2.1. Cadmium Source and Fate in Environment

Cd, rarely existing in its pure form, is commonly associated with zinc sulfide, lead and copper ores. Natural sources of Cd include volcanic activity and the weathering of parent rocks [6]. Anthropogenic sources, such as the use of phosphate fertilizers and soil amendments, wastewater irrigation, smelting, deposition of airborne Cd from mining, and fossil fuel combustion, also contribute to the elevation of soil Cd [7], which has been demonstrated in many regions of China with extensive nonferrous metal mining, smelting, and other related industrial operations [8].

The mobility and bioavailability of Cd in soil are influenced by a variety of factors, including soil organic matter (SOM) and soil pH. SOM immobilizes Cd through the formation of large, stable negatively charged interfaces, thereby decreasing phyto-availability to plants [9]. However, SOM, such as fulvic/humic acids and dissolved organic carbon (DOC), can form soluble chelates with Cd and increase its mobility under certain soil conditions [10]. The application of dihydrazone (DASH), a dialdehyde starch, to Cd-contaminated soil enhanced the phytoextraction of Cd by maize [11].Therefore, amended organic materials have been utilized in controlling the bioavailability of soil Cd either by decreasing crop accumulation or by increasing the extraction of hyperaccumulators [12]. Soil pH, another key factor, has a fundamental impact on Cd mobility and bioavailability [13], mainly because of the competition between Cd and H^+^ for adsorption sites [14]. Generally, the bioavailability of Cd is restrained when soil pH is higher than 6.5, since it leads to an increase in Cd adsorption on the negatively charged soil surface.

### 2.2. Migration Pathways of Cadmium in Plants

#### 2.2.1. Cd in Rhizosphere

Plant roots significantly influence the environment of the rhizosphere, and therefore impact their uptake of Cd. Generally, organic acid excreted from the roots can change Cd solubility via chelation and ligand exchange reactions [15]. Furthermore, by means of proton excretion, the rhizosphere is acidified and can promote the release of Cd from the solid phase. Numerous studies have demonstrated the positive role of organic acids in Cd bioavailability. For example, a significant correlation between oxalic acids and Cd accumulation was observed in rice under Cd-contaminated conditions [16]. Blocking the oxalate secretion of tomato roots by Phenylglyoxal aggravated the Cd toxicity, suggesting that oxalate exudation contributes to Cd resistance in tomato [17]. High excretion of oxalate from the root of *Sedum alfredii* Hance potentially enhanced Cd uptake and accumulation [18]. Other negatively charged anions, such as citric, malic and acetic acid, have also been shown to be incapable of forming stable Cd complexes, which influences plant Cd uptake [19]. Under iron (Fe) deficiency, Cd stress caused greater phytosiderophores production by maize roots, but this release failed to protect maize plants from Cd toxicity [20,21].

Furthermore, different nitrogen forms differentially affect Cd uptake and accumulation. Compared with NO_3_^-^ fertilization, NH_4_^+^ fertilization caused a significant enhancement of soil acidity, which was due to the proton release resulting from the absorption of NH_4_^+^ by the plant roots. The soil acidity concurred with a significant increase in Cd uptake [14].

#### 2.2.2. Root Morphology

Cd assimilation is based on the root morphological structure [22], surface area [23], physiological characteristics [24], and plant growth stages [25]. For example, maize plants with a greater root average diameter inhibit more Cd uptake [26]. Generally, Cd influx is much higher at the root tip than at the root base, as has been observed in wheat [27], sunflower [28], rice [29], etc. Hence, plants with fewer tips exhibit lower Cd translocation [30], which can be used for pre-screen low-Cd-accumulating cultivars [31]. This may be due to the elevated activity of transport systems close to the root tip. Incomplete development of apoplastic barriers near the root apex may also contribute to the higher influx of Cd, as it may favor apoplastic Cd uptake [22]. Based on the modeling analysis conducted by Laporte et al. [32], the total surface area of the root may be a more influential parameter in determining the extent of Cd uptake by the root system.

#### 2.2.3. Cell Wall

As the outermost structure of plant cells, the cell wall functions as the first line of defense against Cd invasion. The negatively charged sites on cellulose, hemicellulose, and pectin chains in plant cell walls allow for the absorption of Cd. Energy-dispersive X-ray micro-analysis (EDX) in the root cortex of *Arabidopsis thaliana* revealed that Cd accumulates in the cell walls together with phosphate ions [33]. In contrast, in the central cylinder of the root, Cd was found to be present as Cd/sulfur (S) granular deposits in the middle lamella of the pericycle, suggesting that Cd may also form complexes with sulfur-containing biomolecules or proteins in this region [33]. The binding capacity of the cell wall for Cd varies depending on the plant species and the specific structural characteristics of the cell-wall matrix. In leaves of oilseed rape, only a small fraction (11%) of the Cd accumulated in cell walls [34], indicating that other cellular compartments, such as vacuoles or organelles, may play a more significant role in Cd sequestration in this species. In *S. alfredii*, more than 60% of Cd was found in the cell-wall fraction [35]. In rice, 70–90% of the total root Cd was found in the cell walls [36].

Under Cd stress, the proportion of cell-wall components and their binding capacity to Cd are commonly altered. Reactive oxygen species (ROS) production induced by Cd stress impacts the cell-wall composition through the regulation of the gene expression of cinnamyl-CoA reductase and cinnamyl alcohol dehydrogenase, promoting pectin biosynthesis and demethylation [37]. This molecular regulation augments the number of functional groups in pectin, such as the hydroxyl and carboxylic groups, enhancing its binding capacity to Cd and initiating xylem development procedures [37,38]. It was found that Cd stress in rice roots triggers the production of H_2_O_2_, which promotes the biosynthesis of pectin. Through demethylation, pectin releases -OH and -COOH, enhancing the binding of Cd to pectin components in root cell walls [37].

It should be noted that the method used for the determination of Cd in cell walls in many studies may be questionable. The use of homogenization and fractionation methods in the liquid phase may alter the original distribution of Cd in the plant tissue due to diffusion or release of the metal from one fraction to another. This can lead to an overestimation or underestimation of the amount of Cd present in each fraction and inaccuracies in the interpretation of Cd distribution in plant cells. Therefore, alternative methods, such as cryo-sectioning [39] or laser microdissection [40], are recommended because they can preserve the integrity of plant cells and avoid the diffusion of metals during the extraction process.

#### 2.2.4. Transporters

**Figure 1:**
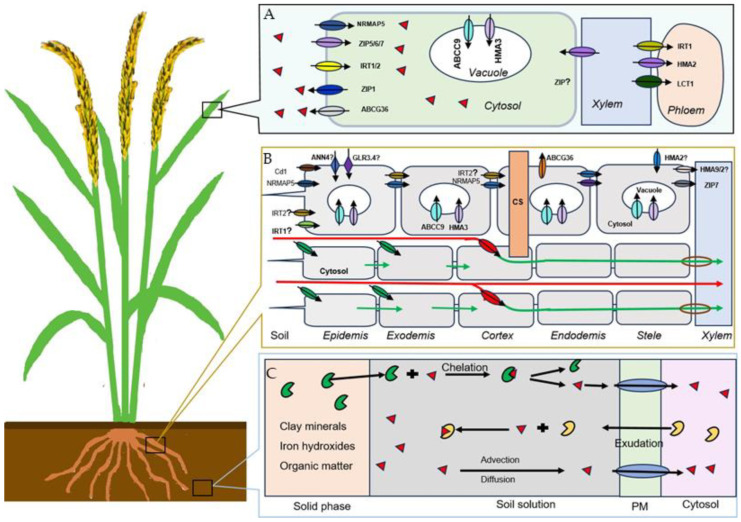
Pathways of Cd in soil-plant system: (**A**) Cd distribution in leaves through transporters. The red triangle represents Cd ion; (**B**) Cd distribution in roots through transporters. The green and red arrows indicate symplastic and apoplastic pathways, respectively. CS: Casparian strip; and (**C**) Cd bioavailability in the range of plant–soil interface. Yellow shape and green shape represent Cd-organic ligands from soil to roots and Cd-organic ligands secreted from roots, respectively. PM: Plasma membrane. For simplification, the cell wall is not presented.

Understanding the transporter protein families of Cd is crucial for developing strategies to reduce Cd accumulation in crops. Several transporter protein families have been identified, including natural resistance-associated macrophage protein (NRAMP), zinc- and iron-regulated transporter protein (ZIP), heavy metal-transporting ATPases (HMA), ATP-binding cassette (ABC), and H^+^/cation-antiporters (CAXs) families, etc. (See Figure 1).

NRAMP proteins are widely present in plants, and mainly function in the transport of Cd and other metal ions, such as Fe, manganese (Mn), aluminium (Al), etc. This gene was first reported in the model plant Arabidopsis, while it was mainly studied in rice among the food corps. *OsNRAMP1*, a transporter localized in the plasma membrane mediating xylem loading, is mainly expressed in the roots. By heterologous expression of *OsNRAMP1* in Arabidopsis, *OsNRAMP1* increased the accumulation of As and Cd [41]. Knockout of *OsNRAMP1* resulted in decreasing Cd uptake by the rice roots and the accumulation in the leaves and grains, while overexpression of *OsNRAMP1* in rice reduced Cd accumulation in the roots, but increased it in the leaves [42,43]. *OsNRAMP2* mediated Cd efflux from the vacuoles in the vegetative tissues, and it was noted that knockout of *OsNRAMP2* significantly decreased the Cd content in rice grains [44]. *OsNRAMP5*, localized at the distal part of the exodermis and endodermis of root cells, is accountable for the influx of Mn and Cd into root cells from external solutions [45].

Several types of ZIP proteins were identified, each with a different role in regulating Cd transport. *OsZIP1*, mainly expressed in the endoplasmic reticulum and the plasma membrane of roots, functions in the Cd efflux transporter. Overexpression of *OsZIP1* resulted in the accumulation of zinc (Zn), copper (Cu), and Cd in rice plants [46]. *OsZIP9* had influx transporter activity that functioned synergistically in the Cd/Zn uptake of rice [47]. Knockout of *OsZIP7* resulted in Cd retention in rice roots, hindering Cd upward transmission and xylem loading and delivery of Cd into the rice grains [48].

HMA transporters, mainly localized on the plasma membrane and tonoplast, regulate the uptake and translocation of Cd through the roots and shoot tissues. For instance, *OsHMA2* is involved in Cd across the cell membrane and in root to shoot translocation [49]. *OsHMA3* regulates the sequestration of Cd in vacuoles to limit the accumulation of Cd in the cytosol [50]. *OsHMA9* is mainly expressed in the root epidermis and outer cortical cells where it functions to transport Cd out of the root for sequestration or storage [51].

ABC transporters are one of the largest known superfamilies, with over 120 members in both Arabidopsis and rice plants. They play a crucial role in the transport of a wide range of substances across membranes, including Cd. In rice, *OsABCC9* is predominantly expressed in the root stele, mediating Cd accumulation by sequestering Cd into the vacuoles [52]. *OsABCG36* is localized in the plasma membrane, and functions as a Cd extrusion pump. Knockout of it induced significantly higher Cd accumulation in root cell sap and significantly increased rice sensitivity to Cd [53].

Cd can also be translocated into the vacuole by CAXs by using the proton gradient to mediate Cd storage in the vacuole of plant cells. Detailed information will be expanded on in the following Ca/Cd section.

#### 2.2.5. Translocation

By means of the Cd transporters, Cd can cross the exodermis and/or the endodermis cell layers within the symplastic route, where the apoplastic route is blocked by the barriers, such as the Casparian strip [22]. However, Cd may also cross into the xylem via the apoplastic pathway where the barrier is incomplete or lacking, such as in the root tip, and in emergence sites of the lateral root and stage I endodermis [54] (See Figure 1).

After being taken up by the roots and loaded into the root xylem, Cd is transported to the above-ground plant parts through the xylem sap flow, which is primarily driven by plant transpiration and the water potential gradient between the soil and atmosphere. Numerous studies have confirmed this mechanism. Spraying a transpiration inhibitor, such as abscisic acid (ABA), on leaves, dramatically reduced Cd accumulation in lndian Mustard leaves [55]. Using isotopic analysis, it was found that in wheat roots, higher transpiration rates were positively related to higher Cd accumulation [56]. Through a determination of Cd levels among 69 rice genotypes, it was found that Cd levels in the xylem sap were strongly correlated to the Cd concentrations in shoots and grains [57].

After long-distance transport, Cd is unloaded from the xylem vessels, which may be similar to the nutrient process via a symplastic pathway. This process occurs through a high branch network of veins that cross the leaf blade. However, there is limited knowledge regarding the transport mechanisms accountable for unloading Cd ions from the xylem. Cd may enter the leaf cells through nutrient transport proteins, such as Fe, Mn, and Zn, which is equally what happens during root uptake. However, the roles in the transporting pathways to the epidermis, and the storage and distribution of Cd in the shoot still remain unknown [58].

#### 2.2.6. Cd Redistribution

Phloem determines the Cd redistribution between the aerial parts of the plant, particularly in the sink organs such as those from leaves into seeds. For example, it has been observed that 91% to 100% of Cd accumulated in rice grains is transported via the phloem [59]. The Cd content in rice grains was correlated with the Cd level of phloem sap, but not with the concentrations in xylem sap [60]. Drawing on the example of Ca as a benchmark for phloem transportation, it can be inferred that phloem served as the principal conduit for Cd transport to sunflower seeds [61]. Similarly, in wheat, 50–60% Cd in mature grains was found to have occurred by re-mobilization through phloem from leaves and stem [62]. Rice nodes, especially the first node, are the important transfer stations where Cd can be loaded into the rice phloem [63]. In addition, it was suggested that *OsHMA2*, highly expressed in the nodes and close to the vascular bundles, could be involved in mediating Cd transference from the xylem sap to the phloem [64].

### 2.3. Cadmium Toxicity to Plants

The toxic mechanisms of Cd in plants are proposed as the following: (1) an imbalance of nutrient uptake resulting in reduced absorption at the root surface; and (2) direct combination with the sulfhydryl (-SH) group, which impairs protein structure thereby interfering with various physiological processes, such as respiration [65], photosynthesis [66], cell division [67], and ROS production/scavenging [68]. Since a number of review papers have fully discussed the toxic mechanisms of Cd [69,70,71,72,73], some hot topics related to Cd–plant research are highlighted.

#### 2.3.1. Hormesis of Cd on Plant Growth

High levels of Cd exposure to plants were shown to cause necrotic lesions, leaf chlorosis, inhibition of root elongation, wilting, reduced biomass, and potential death [74,75,76]. Cd also negatively affects seed germination, but it can be reversed after rinsing, indicating the Cd toxicity was due to seeds not achieving sufficient water rather than as a result of direct phototoxicity [77].

Interestingly, a few studies found that seed germination can be stimulated by low concentrations of Cd stress [78]. Recent evidence for the hormesis of Cd on plant growth has been rapidly accumulating. For instance, a 5 mg kg^−1^ Cd treatment increased the biomass, height, and chlorophyll content in *Lonicera japonica* Thunb, a Cd-hyperaccumulator [79]. A similar finding was also observed in *Polygonatum sibiricum* under 1 mg Cd kg^−1^ [80] and in tomato under 6.9 mg Cd kg^−1^ stresses [81]. The hormesis induced by low-dose Cd stress might be attributed to the overproduction of ROS that intensifies the signaling role in cell-cycle activity [82]. However, the underlying effects on plant metabolism remain insufficiently studied.

#### 2.3.2. Ionomics of Cd with Elements

The assimilation of plant nutrients is greatly interfered with by Cd toxicity. To date, Cd interactions with essential mineral elements, including nitrogen (N), phosphorus (P), potassium (K), silicon (Si), magnesium (Mg), S, Zn, Fe, Ca, boron (B), Mn, Cu, selenium (Se), etc., have been intensively investigated [83]. Under Cd stress, the reduced uptake of nutrients is mainly due to the inhibition of transporters responsible for loading elements into the aerial parts of plants [84]. As mentioned above, the function of co-transporters such as NRAMPs for Fe/Mn/Cd, HMAs for Cu/cobalt (Co)/Zn/Cd, ZIPs for Fe/Zn/Cd, and CAXs for Ca/Cd, will continue to be unveiled. This is a promising field for ionomics research that involves the comprehensive analysis of the elemental composition of biological systems, providing a powerful tool for understanding the impact of Cd toxicity to plants.

In addition to general competition, some elements have specific mechanisms that help reduce the toxicity of Cd in plants. For example, Fe can form a layer of Fe oxide on rice roots, known as iron plaque, to sequester Cd and reduce its bioavailability to rice plants [85]. Moreover, it can induce the synthesis of metallothioneins (MTs), which are small, cysteine-rich proteins that bind to and detoxify Cd [86]. For Si and B, they can promote the deposition of Cd in cell walls, thereby limiting its translocation from roots to shoots by creating a barrier in the endodermis [87,88]. For S, it can reduce the toxicity of Cd in plants by forming thiol compounds such as glutathione (GSH) and phytochelatins (PCs) that help sequester and detoxify Cd [89]. It can also modify the physicochemical properties of the rhizosphere, which affects the availability and mobility of Cd in soil and, consequently, its uptake by plants [90]. Furthermore, Ca has been shown to interact with Cd in various ways, as detailed in the following sections.

#### 2.3.3. Detoxication of Cd by Glutathione

The primary toxicity of Cd in plants is the induction of ROS production, leading to oxidative damage [91]. Although Cd does not directly participate in cellular redox reactions, it disrupts electron transport, damages antioxidant enzyme structures, and interferes with antioxidant molecule synthesis, leading to elevated ROS levels in the cell.

Cd induces ROS production in plants, causing oxidative damage. Among the antioxidant molecules, glutathione (g-Glu-Cys-Gly, GSH) is one of the most important reducing equivalents, protecting plants against Cd-induced oxidative damage. Furthermore, it is also a key molecular compound or a basic component of PCs involved in Cd chelation and thereby confines Cd to less sensitive organelles, such as vacuoles [92]. An increase in the demand for Cd detoxification usually leads to rapid depletion of GSH levels and a loss of antioxidative defense [93]. Numerous studies were conducted on the antioxidative and chelating roles of GSH under Cd stress, and the signaling pathways that regulate these two roles are comprehensive and not well studied.

A number of genetic reports have demonstrated possible links between phytohormone signaling and GSH metabolism. For instance, the *cat2* Arabidopsis mutant, which has high levels of salicylic acid (SA), shows increased GSH levels and SA-dependent responses [94]. Conversely, the SA-deficient mutant *sid2* had much lower GSH levels than wild-type plants [95]. This signaling role of SA may be related to the production of GSH by serine acetyltransferase (SAT) and the recovery of GSH by glutathione reductase1 (GR1) [95]. Furthermore, ethylene signaling was found to be involved in GSH biosynthesis. Arabidopsis roots can produce ethylene, which activates ethylene signaling in leaves and induces GSH biosynthesis in response to Cd stress [96]. In another study, the accumulation of endogenous jasmonic acid (JA) in Cd-stressed *Lycium chinense* plants affected the expression of glutathione reductase (GR), a key enzyme in GSH accumulation and Cd tolerance [97]. Similarly, auxin was shown to activate glutathione-S-transferase (GST) in barley roots under Cd stress [98]. Understanding these signaling pathways can help in the development of strategies to enhance the antioxidant capacity of cells and prevent Cd-induced toxicity.

## 3. Mechanisms of Ca-Mediated Restriction in Cd Translocation in Rice

Calcium, one of essential element for plants, is required in relatively large quantities (0.1–5%) because it is involved in a multitude of structural and biochemical functions, such as cell-wall development, membrane function, enzyme activation, signal transduction, stomatal regulation, and nutrient uptake, etc. [5,99]. It also plays a critical role in protecting plants against various abiotic stresses. Ca helps to maintain ion homeostasis by regulating ion channels and transporters in cell membranes [100]. Ca also regulates the production and scavenging of ROS, reducing oxidative stress [101]. In addition, Ca signaling can activate various stress-responsive proteins that help plants to prevent abiotic damage [102]. Besides of the general protecting roles as mentioned above, Ca displays some special resistant mechanisms when plants are exposed to Cd stress.

### 3.1. Liming

Lime additions are an efficient and cost-effective way to reduce the bioavailability of Cd in soil. Since the pH of lime is much higher than the soil, amending with lime enhances the OH^-^ in soil solutions, leading to the chemical precipitation of Cd(OH)_2_. Furthermore, the reduction of H^+^ increases the ion adsorption sites of soil surface, which slows down the mobility of Cd ions in soil solutions. In southern China, the recommended application rate of CaO is between 0.75 t ha^−1^ to 1.50 t ha^−1^ before soil tillage, resulting in a soil pH increase of 0.50 unit and a significant decrease of 35% in Cd concentrations in rice grains [103]. However, the effectiveness of CaO additions may not be consistent due to the relatively small amounts used, which can make it difficult to distribute uniformly on the topsoil. As an example, in a study by Wang et al. [104], only a modest increase of 0.28 units in soil pH and a 15% decrease in grain Cd were observed, the effects of which were much lower than that of similar doses used by Zhu et al. [103]. Pot experiments have shown more significant changes in soil pH due to liming than field experiments, which may be due to better control of cultivation conditions [105].

Different types of lime, such as burnt lime (CaO), hydrated lime (Ca(OH)_2_) and limestone (CaCO_3_) have different chemical properties that determine their corresponding recommended doses, which vary greatly, ranging from 0.50 t ha^−1^ to 180 t ha^−1^ [106,107]. CaCO_3_ has a lower effect on increasing soil pH compared to Ca(OH)_2_ and CaO. Thus, the recommended amount of CaCO_3_ is usually higher than that of Ca(OH)_2_ or CaO [105]. However, some studies have observed that the lime effect on soil pH was independent of the amount added, potentially due to soil buffer capacity [106]. Soil properties, such as pH, soil organic matter (SOM), cation exchange capacity (CEC), and clay content, typically relate to the buffering capacity, and it is important to carefully compare and quantify the type and amount of lime based on soil conditions [105].

### 3.2. Iron Plaque

Adapted to grow in flooded environments, rice is capable of delivering oxygen to its roots to support respiration. The excess oxygen in the roots is discharged from aerenchyma, oxidizing Fe^2+^ in submerged soils to Fe^3+^ oxides, resulting in a reddish-brown precipitate on the root surface. This is called iron plaque, an amphoteric colloid with a strong physical and chemical adsorption capacity that can affect nutrient and metal uptake by plants [108]. Some studies have shown that iron plaque effectively sequesters Cd from the surroundings, reducing its mobility and bioavailability to plants [109]. For example, a hydroponic research study revealed that the formation of iron plaque on rice roots reduced Cd concentrations in the root by 34% [110]. The decline in Cd accumulation in rice grains was linked to the enhanced formation of ion plaque on the root surfaces [111]. However, the barrier effect of ion plaque has a threshold based on its thickness. When the adsorbed Cd reaches a certain level, it may penetrate the root and cause Cd accumulation in rice plants. Several studies have suggested that this threshold can range from 20 to 27.3 g kg^−1^, with the average being about 23.5 g kg^−1^ [112,113].

As a bivalent ion, Ca is commonly used in exchange adsorption studies because it is abundant in soils and can readily exchange with other ion cations. For instance, the CaCl_2_ solution ranged from 10 to 100 mM has been widely utilized as an extractor for soil-available Cd [114]. A 5 mM CaCl_2_ solution could even recover 99% Cd from the extraplasmic bodies of wheat roots [115]. However, a previous study indicated that exogenous Se^4+^ and Se^6+^ solutions failed to affect the adsorption of Cd on iron plaque, possibly due to the different valence states between Se and Cd [116]. Since both Cd and Ca are bivalent ions with similar ionic radii, the desorption of Cd on the iron plaque by exogenous Ca may play an important role in preventing Cd translocation in rice roots. However, this hypothesis remains unconfirmed.

### 3.3. Cell-Wall Synthesis

Recently, there has been a growing interest in using nutrient elements as exogenous substances to reinforce the cell-wall structure and prevent Cd migration in cells. Silicon (Si), for example, mainly accumulates in cell walls in the form of a wall-bound organosilicon compound. During in situ examination of cellular fluxes of Cd in suspension cells, it was observed that cells treated with Si exhibited a significant inhibition of net Cd influx compared to cells without Si treatment [117]. A signal investigation revealed that K reduced the expression levels of brassinolide synthesis genes in Cd-stressed *Panax notoginseng* (Burk.) roots. As a result, the biosynthesis of brassinolides was hindered, leading to a reduced expression of the pectin methylesterase gene (PME) and then caused an increase in pectin methylation, which ultimately results in reduced Cd accumulation [118]. Applying boron (B) to the roots increases pectin content by modifying biosynthesis pathways, inhibiting pectinase activity, and reducing the expression levels of associated genes. This leads to an increase in chelation of Cd onto cell walls and a decrease in Cd uptake by organelles via enhanced pectin demethylation. B application also normalizes the levels of cellulose and hemicellulose in the cell walls and enhances gene expression from the expansion, xyloglucan endotransglucosylase, and a-xylosidase families, thus strengthening cell-wall integrity and root flexibility. As a result, the accumulation of reactive oxygen species (ROS) is curbed and damage to the root surface structure is mitigated, leading to an increase in root viability [119].

As an essential nutrient element for plants, Ca plays a significant role in maintaining the structural stability of plant cell walls. Through the binding of galacturonic acid residues, Ca forms a pectin calcium gel, which connects adjacent cells and increases cell toughness as a component of both the cell wall and intercellular layer [120]. Spraying CaCl_2_ onto grapevines resulted in the downregulation of the PG1 and PG2 genes encoding polygalacturonase, while the cellulose synthase family gene CesA3 in grape peel was unaffected. These findings highlight the vital role of Ca in inhibiting the degradation of pectin components and stabilizing the structure of the cell wall [121].

The Ca^2+^ signaling pathway in plants is intricately linked to active oxygen metabolism [122] in a process called ROS-mediated Ca^2+^ signaling. Exogenous Ca prevented the accumulation of superoxide radicals induced by Cd in mesophyll cells of pea plants, suggesting Ca regulates the cellular response to the Cd exposure [123]. Low ROS levels stimulate Ca^2+^ channels, allowing for a rapid influx of Ca^2+^ into the cytosol of the cell. This influx then triggers the activation of downstream signaling pathways, which can lead to a range of physiological responses, such as regulation of the synthesis of cell-wall components, particularly pectin. However, under conditions of high oxidative stress, excessive ROS production can overwhelm the Ca^2+^ signaling pathway, leading to the loss of Ca^2+^ homeostasis and negative impacts on cell viability [124,125]. To date, research on the interplay between Ca/Cd in the synthesis and modification of cell-wall components relating ROS production has been relatively limited.

### 3.4. Calcium Carrier Proteins (CAXs) Family

Cd is a non-essential element in plants, and its active transport is mainly facilitated by divalent cation transporters with relatively low specificity, such as Zn transporter (ZRT), Fe transporter (IRT), and Fe/Mn/Zn cotransporter (NRAMP), etc. Cd^2+^ and Ca^2+^ have comparable ionic radii, which leads to competition between these two elements for ion channels and carrier proteins on the root surface of plants. This competition can inhibit Ca absorption by plants. For example, Cd induces the depolarization of wheat root tip cells, resulting in a decrease in the amount of Ca adsorbed by the cells, and a subsequent reduction in the net content of Ca in the root [126]. The addition of La^3+^ (a Ca channel inhibitor) to an *S. alfredii* suspension cell system showed significant inhibition of Cd transport to protoplasts [127].

The Ca^2+^/H^+^ reverse carrier protein family, also known as CAXs, is an ion channel protein that facilitates Ca/Cd co-transport. The ATPase on the vacuolar membrane generates an H^+^ electrochemical potential gradient, which drives the CAXs function. Cooperation between CAXs and the HMA protein family is responsible for transporting Cd from the cytoplasm to the vacuole [128], and achieving Cd segregation. The N terminus of CAXs contains an autoinhibitory region, while two conserved regions (c-1 and c-2) are primarily responsible for ion selectivity. The difference in these regions determines which ions the CAXs family members can select [129].

Currently, CAX family genes involved in Cd uptake and transport have been cloned and identified in different plants. However, their capacity for Cd transport remains a point of debate. Arabidopsis, for instance, has six AtCAXs family members, and it was previously suggested that AtCAX2 and AtCAX4-coded proteins have the highest ability to transport Cd [130]. However, recent quantitative trait loci (QTL) mapping studies reveal that a loss of AtCAX1 gene function leads to the high sensitivity of Arabidopsis to Cd toxicity [131]. Similarly, the rice OsCAXs family has six members, namely OsCAX1a, OsCAX1b, OsCAX1c, OsCAX2, OsCAX3 and OsCAX4 [132]. Among them, the loss of OsCAX2 function resulted in increasing Cd toxicity, while the upregulation of its expression significantly inhibited Cd accumulation in rice [133]. Preliminary evidence from a yeast heterologous system showed that OsCAX1a, OsCAX1c and OsCAX4 had Cd transport activity in rice [132]. However, the extent to which each member of the OsCAX family can transport Cd, and their potential synergistic effects, remain subjects for further investigation.

### 3.5. Transpiration

Cd translocation from root to shoot occurs mainly via two routes: the apoplast (xylem-passive transport) and symplastic (phloem-active transport) routes. The apoplast route, relying on transpiration, accounts for over 90% of Cd transport [55]. Thus, all the external factors that influence plant transpiration (e.g., temperature, light, ABA, transpiration inhibitors, etc.) affect Cd transport to the shoot [134]. For example, when the leaves of India mustard were subjected to 100 M ABA for 24 h, stomatal diffusion resistance increased by nearly 50 times, leading to a substantial decrease in transpiration rate. This caused Cd transport to the shoot to nearly cease, possibly due to the Ca^2+^ signal transduction [55]. Shading significantly reduced the transpiration rate in tobacco leaves, leading to a 73.5% decrease in Cd accumulation [134]. Similarly, shading rice leaves by intercropping Sesbania significantly inhibited both transpiration and Cd translocation in rice grains [112].

Similar to Cd, Ca transport in plants also occurs mainly through the apoplast pathway with water serving as the carrier. To facilitate the stable function of Ca^2+^ signals, plants have evolved regulatory pathways for water transport (transpiration) in response to changes in Ca^2+^ concentration. The process may involve the reverse regulation of aquaporins (AQPs) in leaf guard cells. When the cytosolic Ca^2+^ content is low, AQPs are activated and Ca^2+^ from the apoplast, along with water, flow into the cytoplasm. Conversely, when the cytosolic Ca^2+^ content is high, AQPs shut down and reduce the hydraulic conductivity of leaves to prevent excessive Ca^2+^ accumulation in the cytoplasm [135]. Thus, the Ca ion can be utilized as a transpiration regulator for plants; a previous study observed that spraying CaCl_2_ on tobacco leaves significantly reduced transpiration and Cd accumulation. However, the underlying mechanism has not been thoroughly investigated [134].

## 4. Conclusion and Perspectives

In recent decades, the cycle of Ca through the soil and ecosystem has been significantly affected by rising temperatures and changes in precipitation patterns. Increased temperatures speed up the decomposition of organic matter in soils, alongside Ca release from the soil, making it less available to plants. In addition, droughts, dry spells, and heavy rainfall events lead to Ca leaching, as soil water moves through the soil and carries the nutrient away. The loss of Ca has far-reaching consequences for ecosystems. Plants may experience Ca deficiency, which reduces their growth, productivity, and resistance to abiotic stresses, including Cd toxicity. For this reason, the possible roles of Ca in mitigating Cd translocation in rice were reviewed here. To decrease grain Cd concentrations without compromising grain yield, two effective and low-cost methods were suggested for rice producers: (1) raising soil pH to 6.5 with the application of Ca compounds before rice seedling transplantation [136]; and (2) spraying Ca solutions on the rice leaves in the booting stage.

**Figure 2:**
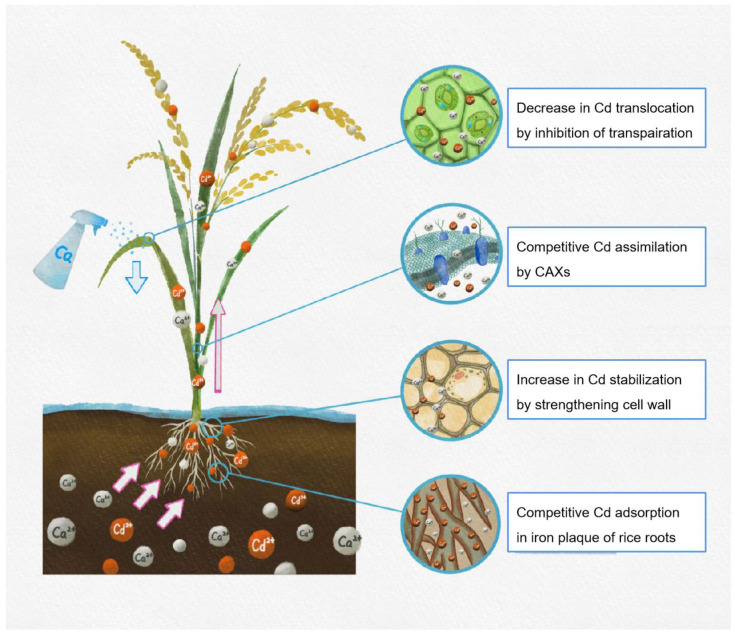
Possible role of inhibition effect of Ca on the Cd translocation in rice.

Ca is known for its role in cell-wall composition, transporter gene expression, and transpiration, which have a crucial role in Cd tolerance. Hence, the special proposed mechanisms in this review included desorption of Cd on the iron plaque of rice roots, maintaining the structural stability of the cell wall, co-transport of Cd by CAXs, and inhibiting Cd translocation by regulating transpiration (see Figure 2). In addition to the aforementioned mechanisms, several general functions concerning ion homeostasis, ROS regulation, and the synthesis of stress-responsive proteins should also be further elaborated on. Furthermore, an increasing number of studies indicate that the function of Ca is mediated by signaling messengers, such as plant hormones, nitric oxide (NO), and ROS. Thus, the crosstalk between Ca and signaling messengers may be an important research topic in the mechanisms of Ca-mediated restriction in Cd translocation in plants.

## Data Availability

Not applicable.

## Abbreviations

ABA	Abscisic acid
ABC	ATP-Binding Cassette
Al	Aluminium
AQPs	Aquaporins
B	Boron
Ca	Calcium
CAX	H^+^/cation-antiporters Exchanger
Cd	Cadmium
CEC	Cation exchange capacity
Co	Cobalt
CS	Casparian strip
Cu	Copper
DASH	Dihydrazone
DOC	Dissolved organic carbon
EDX	Energy-dispersive X-ray micro-analysis
Fe	Iron
GR	Glutathione reductase
GR1	Glutathione reductase1
GST	Glutathione-S-transferase
HMA	Heavy Metal-transporting ATPases
JA	Jasmonic acid
K	Potassium
Mg	Magnesium
Mn	Manganese
MTs	Metallothioneins
N	Nitrogen
NO	Nitric oxide
NRAMP	Natural Resistance-Associated Macrophage Protein
P	Phosphorus
PCs	Phytochelatins
PM	Plasma membrane
PME	Pectin methylesterase gene
QTL	Quantitative Trait Loci
ROS	Reactive oxygen species
S	Sulfur
SA	Salicylic acid
SAT	Serine acetyltransferase
Se	Selenium
Si	Silicon
-SH	Sulfhydryl
SOM	Soil organic matter
ZIP	Zinc and Iron regulated transporter
Zn	Zinc
